# *Astragalus membranaceus* formula for moderate-high risk idiopathic membranous nephropathy: A meta-analysis

**DOI:** 10.1097/MD.0000000000032918

**Published:** 2023-03-03

**Authors:** Dan Wang, Lijuan Wang, Mingrui Zhang, Ping Li, Qinghua Zhang, Kun Bao

**Affiliations:** a Second Clinical Medical College, Guangzhou University of Chinese Medicine, Guangzhou, China; b The Second Affiliated Hospital of Guangzhou University of Chinese Medicine, Guangdong, China; c Guangdong-Hong Kong-Macau Joint Lab on Chinese Medicine and Immune Disease Research, Guangdong, China; d State Key Laboratory of Dampness Syndrome of Chinese Medicine, The Second Affiliated Hospital of Guangzhou University of Chinese Medicine, Guangdong, China.

**Keywords:** *Astragalus membranaceus*, Chinese medicine, Idiopathic membranous nephropathy, meta-analysis, systematic review

## Abstract

Idiopathic membranous nephropathy (IMN) is a noninflammatory autoimmune glomerulonephropathy. Based on the risk stratification for disease progression, conservative nonimmunosuppressive and immunosuppressive therapy strategies have been recommended. However, there remains challenges. Therefore, novel approaches to treat IMN are needed. We evaluated the efficacy of *Astragalus membranaceus (A membranaceus*) combined with supportive care or immunosuppressive therapy in the treatment of moderate-high risk IMN.

**Methods:**

We comprehensively searched PubMed, Embase, the Cochrane Library, the China National Knowledge Infrastructure, the Database for Chinese Technical Periodicals, Wanfang Knowledge Service Platform, and SinoMed. We then performed a systematic review and cumulative meta-analysis of all randomized controlled trials assessing the two therapy methods.

**Results:**

The meta-analysis included 50 studies involving 3423 participants. The effect of *A membranaceus* combined with supportive care or immunosuppressive therapy is better than that of supportive care or immunosuppressive therapy along in regulating for improving 24 hours urinary total protein (MD = −1.05, 95% CI [−1.21, −0.89], *P* = .000), serum albumin (MD = 3.75, 95% CI [3.01, 4.49], *P* = .000), serum creatinine (MD = −6.24, 95% CI [−9.85, −2.63], *P* = .0007), complete remission rate (RR = 1.63, 95% CI [1.46, 1.81], *P* = .000), partial remission rate (RR = 1.13, 95% CI [1.05, 1.20], *P* = .0004).

**Conclusions:**

Adjunctive use of *A membranaceus* preparations combined with supportive care or immunosuppressive therapy have a promising treatment for improving complete response rate, partial response rate, serum albumin, and reducing proteinuria, serum creatinine levels compared to immunosuppressive therapy in people with MN being at moderate-high risk for disease progression. Given the inherent limitations of the included studies, future well-designed randomized controlled trials are required to confirm and update the findings of this analysis.

## 1. Introduction

In China, membranous nephropathy (MN) emerged as the leading type of biopsy finding in patients aged > 40 years, which would soon surpass IgA nephropathy.^[1]^ Idiopathic membranous nephropathy (IMN) is a noninflammatory autoimmune glomerulonephropathy, characterized by the formation of immune complex deposits on the subepithelial in the kidney.^[2]^ The most patients with IMN present nephrotic range proteinuria.^[3–5]^ Approximately 40% of patients will undergo spontaneous remission,^[6]^ while another 30% will have a poor response to immunosuppressive therapy and then progress to end-stage renal disease (ESRD).^[7]^ If follow-up is extended to 10 to 20 years, progression to ESRD may occur in 50% to 60% of patients without treatment.^[8]^ Based on the risk stratification for disease progression, conservative nonimmunosuppressive and immunosuppressive therapy strategies have been recommended by the Kidney Disease: Improving Global Outcomes (KDIGO) Clinical Practice Guideline to treat patients with IMN.^[9]^ Unfortunately, there remains challenges of compromised clinical response, high cost, serious adverse effects and high recurrences.^[10–16]^ Therefore, novel approaches to treat IMN are needed.

Over thousands years, traditional Chinese medicine (TCM) has been extensively used in East Asia and developed a unique theoretical system, containing many different therapeutic and preventive methods (such as Chinese herbal medicine, acupuncture and moxibustion).^[17]^ Astragalus (Radix Astragali) is one of the most widely prescribed herbs in traditional Chinese medicine. Astragalus (huang qi in Chinese), is the dry root of *Astragalus membranaceus (A membranaceus*; Fisch.) Bge. var. Mongholicus (Bge.) Hsiao or *A membranaceus* (Fisch.) Bge. Hitherto, over 100 chemical constitutions have been isolated and identified from A.membranaceus, including flavonoids, saponins, polysaccharides, and amino acids.^[18–20]^ To date, a number of studies in animal and cellular models have proven that Astragalus possesses potent protective effects in kidney.^[21–23]^ Many clinical studies have demonstrated that Astragalus can improve kidney function and reduce proteinuria.^[24–26]^ However, the quality of these studies had not been assessed systematically. It is imperative to assess the efficacy and safety of *A membranaceus* as adjunctive therapy to Western medicine therapy for IMN. The aim of the current study is to evaluate the effificacy of *A membranaceus* combined with supportive care or immunosuppressive therapy in the treatment of moderate-high risk IMN.

## 2. Methods

This systematic review followed the methods of the Cochrane Handbook for Systematic Reviews of Interventions (version 6.3) and complied with the 2009 Preferred Reporting Items for Systematic Reviews and Meta-Analyses statement guidelines.^[27,28]^ We registered the review protocol with PROSPERO at the beginning (CRD: 42021232472).

### 2.1. Criteria for considering studies for this review

#### 2.1.1. Types of studies.

Randomized controlled trials (RCTs) on the treatment of adults with IMN using oral Astragalus preparation were eligible. Quasi-RCTS (RCTs in which allocation to treatment was obtained by alternate medical records, alternation, use of date of birth, or other divinable methods) were excluded. There was no restriction on languages or publication status.

#### 2.1.2. Types of participants.

##### 2.1.2.1. Inclusion criteria.

We included adults (aged 18 years and older) histologically diagnosed with IMN. Patients were classified as being at moderate-high risk for disease progression when recruited. According to the KDIGO classification: moderate risk is defined as normal eGFR, proteinuria of more than 3.5 g/d and no decrease of more than 50% after 6 months of conservative therapy with angiotensin converting enzyme inhibitor (ACEi) or angiotensin II receptor blocker (ARB), and not fulfilling high-risk criteria. While high risk is defined as eGFR of less than 60 mL/min/1.73 m^2^ and/or proteinuria of more than 8 g/d for more than 6 months; or normal eGFR, proteinuria of more than 3.5 g/d and no decrease of more than 50% after 6 months of conservative therapy with ACEi or ARB, and at least one of the following: serum albumin of less than 25 g/L; anti-phospholipase A2 receptor antibody of more than 50 RU/mL. Therapeutic regimen complied with KDIGO guideline. Oral forms of *A membranaceus* preparation, including boiled decoction, extracts and granules were eligible.

##### 2.1.2.2. Exclusion criteria.

Studies with the following conditions will be excluded: Trials included patients with secondary forms of membranous nephropathy; Study data could not be available from the report or by contacting the authors; Patients who were received renal replacement therapy; Studies assessed *A membranaceus* combined with other complementary therapies (such as moxibustion, acupuncture); Therapies related to TCM were used in the control group; Western medicine was contained in both groups, but they were differented from each other; Huangqi doesn‘t serves as a principal medicine.

#### 2.1.3. Types of interventions.

All participants received routine therapies according to clinical practice guidelines. Conventional therapy includes more rest, low salt, low fat, high quality and protein diet; drugs aimed to correct dyslipidaemia (e.g., statins), antialdosterone drugs (e.g., spironolactone), antihypertensive (e.g., ACEi or ARB), antithrombotic agents (e.g., dipyridamole).

Treatment group participants needed to have received oral Astragalus formula (decoction, pill, powder, or capsule) in combination with immunosuppressive therapy.

Control group participants received immunosuppressive therapy.

Huangqi serves as a principal medicine, defined as follows: the properties of Huangqi are consistent with the main aims of the formula.

#### 2.1.4. Types of outcome measures.

##### 2.1.4.1. Primary outcomes.

Complete response rate was assessed according to the definition provided in each single study.

Partial response rate was assessed according to the definition provided in each single study.

Proteinuria measured by 24 hours urinary total protein (UTP), Urine protein/creatinine ratio.

Kidney function measured by Serum creatinine concentration (SCr).

Adverse events.

##### 2.1.4.2. Secondary outcomes.

Disease activity assessed by serum albumin.

Primary outcome or secondary outcome measurements were collected immediately after treatment and at the end of follow-up period.

### 2.2. Search strategy

We searched seven databases from their inception to May 2022. Three English databases include EMBASE, MEDLINE, Cochrane Central Register of Controlled Trials. The following Chinese databases were also searched: China National Knowledge Infrastructure, China Biomedical Literature Database, Chongqing VIP, and Wanfang. Reference lists of significant reviews on similar topics and relevant studies were examined.

The search strategies applied for this review were shown (Appendix 1, Supplemental Digital Content, http://links.lww.com/MD/I444).

### 2.3. Data collection and analysis

Two authors independently screened the titles, abstract, and full-text and discarded studies which were not satisfied the eligibility criteria. Then, they in parallel extracted data from eligible studies, using a pre-designed data extraction form. Any published versions discrepancies was highlighted. If more than one publication about one study existed, we grouped them together and used the most complete data. When necessary, original authors were contacted by email to clarify details or to acquire further information about their trials. Information about study characteristics (the first author’s name, participants gender, age, histological type, proteinuria severity, sample size, follow-up period.), intervention protocol of *A membranaceus* (dosage forms, dosage, frequency, and duration), concurrent supportive care or immunosuppressive therapy, and outcome data was collected. Two reviewers assessed the methodological quality of included studies independently, using the Cochrane risk of bias tool (Cochrane Handbook V.5.1.0)^[29]^ (See Table 1). Individual studies were graded as high, unclear, or low risk of bias. Heterogeneity was assessed using the Cochrane Q statistic and *I*^2^ test. A *P* value less than .05 was used for statistical significance.^[30]^ *I*^2^ values more than 50% were correspond to high levels of heterogeneity. Publication bias were examined by using funnel plots for asymmetry and Egger’s linear regression analysis, when one outcome included 10 or more studies. A third author examined the consistency check. If the consensus was not reached, methodologists were consulted to resolve it.

**Table 1:** Characteristics of the included studies.

First author, year [Ref]	Intervention (ingredients of Huangqi formula)	Control participant (Intervention/control)	Participant (Intervention/control)	Age mean ± SD (yr)	Treatment duration (mo)	Complete response	Partial response	Baseline eGFR mean ± SD (mL/min)	Baseline proteinuria mean ± SD (g/24 h)
Caiz 2016	Shenqi Dihuang Decoction (tangshen 15 g, milkvetch root 20 g, prepared rehmannia root 15 g, Chinese yam 15 g, indian bread 20 g, oriental waterplantain rhizome 10 g, tree peony root bark 10 g, asiatic cornelian cherry fruit 15 g)	Angiotensin converting enzyme inhibitor/angiotensin II receptor blocker	22/23	46.36 vs 44.42	6	24 h urine protein of less than 0.5 g, serum albumin of more than 35 g/L, nephritic syndrome disappeared completely.	24 h urine protein of less than 3 g, serum albumin improved.	–	4.63 ± 0.81 4.47 ± 0.7
Caiz 2019	Shenqi Dihuang Decoction (tangshen 15 g, milkvetch root 20 g, prepared rehmannia root 15 g, Chinese yam 15 g, indian bread 20 g, oriental waterplantain rhizome 10 g, tree peony root bark 10 g)	Angiotensin converting enzyme inhibitor/angiotensin II receptor blocker	32/26	42.26 ± 13. 26	6	24 h urine protein of less than 0.5 g, serum albumin of more than 35 g/L, nephritic syndrome disappeared completely.	24 h urine protein of less than 3 g, serum albumin improved.	–	4.99 ± 1.51 4.90 ± 1.03
				44.32 ± 13. 32					
Duohl 2020	Self-formulated Jianpi Lishi Tongluo Prescription (milkvetch root 20 g, indian bread 15 g, oriental waterplantain rhizome 10 g, largehead atractylodes rhizome 12 g, glabrous greenbrier rhizome 10 g, plantain seed 15 g, Chinese waxgourd peel 15 g, cicada slough 10 g, earthworm 10 g, black-tail snake 10 g, tortoise carapace and plastron 9 g, danshen root 10 g, safflower 10 g, figwort root 12 g, dwarf lilyturf tuber 12 g)	Angiotensin converting enzyme inhibitor	36/36	46.2 ± 10.45	3	Proteinuria remained negative or normal 24 h urine protein, normal eGFR.	Sustained 25–50% reduction in urinary protein, normal eGFR.	–	5.64 ± 3.11 5.6 ± 2.4
				47.97 ± 8.87					
Lix 2014	Therapy of Invigorating Spleen and Kidney, Activating Blood and Dispelling Wind (milkvetch root 30 g, cherokee rose fruit 30 g, gordon euryale seed 30 g, coix seed 30 g, largehead atractylodes rhizome 15 g, eucommia 15 g, danshen root 15 g, dodder seed 15 g, stiff silkworm 10 g, tangshen 10 g, Chinese angelica 10 g, peach seed 10 g, divaricate saposhnikovia root 6 g)	Angiotensin converting enzyme inhibitor/angiotensin II receptor blocker	32/31	28.1 ± 7.26	6	Proteinuria remained negative or 24 h urine protein of less than 0.2 g, normal eGFR.	Sustained 25–50% reduction in urinary protein, normal eGFR.	–	4.23 ± 1.22 4.08 ± 1.35
				26.23 ± 6.71					
Mazw 2011	Jia-wei-bu-yang-huan-wu Powder (milkvetch root 60 g, Chinese angelica 12 g, Sichuan lovage rhizome 9 g, earthworm 9 g, peach seed 10 g, safflower 10 g, peony root 12 g, Chinese yam 15 g, asiatic cornelian cherry fruit 12 g, cowherb seed 15 g, plantain seed 15 g, barbary wolfberry fruit 12 g, tangshen 15 g.)	Angiotensin converting enzyme inhibitor/angiotensin II receptor blocker	30/30	46.07 ± 11.06	1	Proteinuria remained negative or 24 h urine protein of less than 0.2 g, serum albumin of more than 35 g/L, normal eGFR.	24 h urine protein of less than 3 g, serum albumin improved.	–	4.96 ± 2.53 4.93 ± 2.25
				48.37 ± 9.70					
Pangzx 2019	Jianpi Bushen Decoction (milkvetch root 30 g, plantain seed 12 g, Chinese yam 12 g, tangshen 12 g, cowherb seed 12 g, Chinese angelica 9 g, peach seed 9 g, asiatic cornelian cherry fruit 9 g, safflower 9 g, barbary wolfberry fruit 9 g, peony root 9 g, earthworm 6 g, Sichuan lovage rhizome 6 g)	Angiotensin converting enzyme inhibitor	52/51	44.10 ± 4.29	1	nephritic syndrome disappeared completely.	Symptoms and signs improved.	–	4.99 ± 2.36 4.98 ± 2.33
				43.91 ± 4.33					
Pinggh 2021	Qiqi Yishen capsule (milkvetch root, asiatic cornelian cherry fruit, Indian bread, coix seed, barbary wolfberry fruit, chinese angelica, Radix Salviae Miltiorrhizae, peony root, twotoothed achyranthes root, rhubarb root and rhizome, yerbadetajo herb)	Angiotensin II receptor blocker	40/40	41.3 ± 10.2	4	Normal 24 h urine protein.	40% reduction in urinary protein.	81.47 ± 15.042 83.24 ± 15.651	5.49 ± 1.13 5.93 ± 0.88
				40.3 ± 11.1					
Panz 2020	Shen Zhi HuoXue Decoction (milkvetch root 50 g, leech 15 g, tangshen 20 g, peach seed 15 g, safflower 15 g, peony root 15 g, dwarf lilyturf tuber 10 g, Chinese angelica 15 g, unprocessed rehmannia root 15 g, lotus seed 10 g, Chinese wolfberry root-bark 10 g, liquorice root 10 g)	Angiotensin II receptor blocker	15/15	48.13 ± 12.12	2	Proteinuria remained negative or normal 24 h urine protein, serum albumin of more than 35 g/L, normal eGFR.	40% reduction in urinary protein.	–	4.11 ± 1.455 4.3 ± 1.355
				41.26 ± 10.79					
Qiaoln 2020	Yiqi Huashi Tongluo Decoction (milkvetch root 30 g, tangshen 15 g, leech 6 g, danshen root 30 g, indian bread 20 g, two toothed achyranthes root 20 g, unprocessed rehmannia root 24 g, zhuling 20 g, oriental waterplantain rhizome 15 g, earthworm 12 g, coix seed 15 g, hedyotis 15 g)	Angiotensin II receptor blocker	30/29	47.27 ± 9.938	3	Proteinuria remained negative or normal 24 h urine protein, normal eGFR.	40% reduction in urinary protein.	–	5.22 ± 1.82 4.91 ± 1.63
				43.07 ± 9.083					
Wangl 2020	Shenqi Dihuang Decoction (milkvetch root 20 g, tangshen 15 g, prepared rehmannia root 15 g, Chinese yam 15 g, asiatic cornelian cherry fruit 15 g, indian bread 20 g, tree peony root bark 10 g, oriental waterplantain rhizome 10 g)	Angiotensin converting enzyme inhibitor/angiotensin II receptor blocker	28/26	47.6 ± 13.2	6	24 h urine protein of less than 0.5 g, serum albumin of more than 35 g/L.	24 h urine protein of less than 3 g, serum albumin improved.	–	5.87 ± 1.21 6.26 ± 1.12
				46.6 ± 12. 9					
Yangfw 2021	Yishen Tongluo Recipe (milkvetch root 20 g, largehead atractylodes rhizome 15 g, tangshen 15 g, epimedium herb 15 g, chinese angelica 15 g, zedoray rhizome 12 g, earthworm 12 g, leech 3 g)	Angiotensin converting enzyme inhibitor/angiotensin II receptor blocker	23/22	46.82 ± 9.43	6	24 h urine protein of less than 0.3 g, serum albumin of more than 35 g/L, normal eGFR.	50% reduction in urinary protein.	–	5.47 ± 1.87 5.32 ± 2.31
				45.63 ± 9.14					
Aiy 2017	Dispersing three jiao-activating blood-dredging collateral Recipe (milkvetch root, cassia twig, epimedium herb, cablin patchouli herb, cardamon fruit, largehead atractylodes rhizome, indian bread, Sichuan lovage rhizome, danshen root, safflower, earthworm, leech)	Prednisone and cyclophosphamide	30/29	45 vs 42	6	Proteinuria remained negative or 24 h urine protein of less than 0.2 g, normal eGFR.	Sustained 25–50% reduction in urinary protein, normal eGFR.	–	5.86 ± 1.5 5.26 ± 0.92
Daim 2018	Qingrehuoxue Hushen Decoction (milkvetch root 30 g, largehead atractylodes rhizome 15 g, atractylodes rhizome 10 g, Chinese yam 20 g, zhuling 12 g, indian bread 12 g, Chinese angelica 15 g, Sichuan lovage rhizome 10 g, barbated skullcup herb 15 g, stiff silkworm 15 g, hedyotis 30 g, coix seed 30 g, earthworm 10 g)	Prednisone and tacrolimus	30/30	--	6	Proteinuria remained negative or 24 h urine protein of less than 0.2 g, serum albumin of more than 35 g/L, normal eGFR.	24 h urine protein of less than 3.5 g, serum albumin improved.	–	5.46 ± 2.02 5.12 ± 1.32
Guowg 2015	Self-formulated Prescription (milkvetch root 60 g, dodder seed 20 g, barbary wolfberry fruit 20 g, ginger processed pinellia 10 g, stiff silkworm 15 g, common buried rubber 15 g, zedoray rhizome 15 g, centipede 2 g)	Methylprednisolone and prednisone and cyclophosphamide	37/37	38.2 vs 37.6	6	24 h urine protein of less than 0.3 g, serum albumin of more than 35 g/L, normal eGFR.	24 h urine protein of less than 3.5 g, serum albumin improved.	–	7.35 ± 2.03 6.86 ± 1.89
Guoyp 2021	Qidi Taozhi Erchan Recipe (milkvetch root 50 g, unprocessed rehmannia root 30 g, peach seed 12 g, earthworm 12 g, leech 9 g, cicada slough 10 g, amur cork-tree 10 g, heterophylly falsestarwort root 15 g, atractylodes rhizome 15 g, indian bread 15 g, epimedium herb 15 g, coix seed 20 g)	Tacrolimus	42/42	46.96 ± 8.63	3	24 h urine protein of less than 0.3 g, serum albumin of more than 35 g/L, normal eGFR.	24 h urine protein of less than 3.5 g, serum albumin improved.	–	4.38 ± 2.79 4.53 ± 2.46
				48.21 ± 7.57					
Haoj 2017	Huangzhiyishen capsule (milkvetch root, leech, barbary wolfberry fruit, Chinese yam, coix seed, sanqi)	Glucocorticoid and cyclophosphamide	32/32	47.72 ± 6.84	6	24 h urine protein of less than 0.3 g, serum albumin of more than 35 g/L, normal eGFR.	24 h urine protein of less than 3.5 g, serum albumin improved.	–	5.72 ± 1.56 5.85 ± 1.84
Hexc 2016	Self-formulated Qingxue Xiaobai Prescription (Chinese yam 15 g, indian bread 15 g, common anemarrhena rhizome 20 g, kudzuvine root 15 g, plantain seed 15 g, milkvetch root 30 g, Chinese magnoliavine fruit 20 g, sanqi 10 g, snakegourd root 15 g, finger citron 20 g, coix seed 20 g)	Prednisone and cyclosporin A	50/50	42.22 ± 3.65	6	24 h urine protein of less than 0.2 g, serum albumin of more than 35 g/L, normal eGFR.	24 h urine protein of less than 3.5 g, serum albumin improved.	–	5.32 ± 0.47 5.43 ± 0.54
Hug 2020	Yishen Huashi Granules (ginseng, milkvetch root, largehead atractylodes rhizome, indian bread, oriental waterplantain rhizome, pinellia tuber, incised notopterygium rhizome and root, doubleteeth pubescent angelica root, divaricate saposhnikovia root, Chinese thorowax root, golden thread, debark peony root, dried tangerine peel, liquorice root, fresh ginger, Chinese date)	Prednisone and tacrolimus/cyclosporin A	21/20	–	9	24 h urine protein of less than 0.5 g, serum albumin of more than 35 g/L, normal eGFR.	Sustained 50% reduction in urinary protein, normal eGFR.	–	–
Jiaozs 2018	ShenQi ZhiLong Decoction (tangshen 20 g, milkvetch root 20 g, leech 6 g, earthworm 10 g, turmeric yellow 10 g, giant knotweed rhizome 15 g, glabrous greenbrier rhizome 15 g, hedyotis 30 g)	Tacrolimus	30/30	45.43 ± 7.25	3	24 h urine protein of less than 0.3 g, serum albumin of more than 35 g/L, normal eGFR.	24 h urine protein of less than 3.5 g, serum albumin improved.	–	4.3 ± 0.39 4.19 ± 0.24
				46.86 ± 6.79					
Ladh 2018	The Decoction of Benefiting Kidney Qi and Promoting Blood Circulation (milkvetch root 30 g, tangshen 15 g, Chinese yam 30 g, indian bread 30 g, asiatic cornelian cherry fruit 15 g, prepared rehmannia root 15 g, dodder seed 15 g, largehead atractylodes rhizome 10 g, danshen root 15 g, Chinese angelica 15 g, gordon euryale seed 30 g, cherokee rose fruit 15 g, dandelion 30 g, Radix Arnebiae 10 g)	Cyclosporin A	46/52	48.15 ± 2.75	2	24 h urine protein of less than 0.3 g, serum albumin of more than 35 g/L, normal eGFR.	24 h urine protein of less than 3 g, serum albumin improved.	–	5.93 ± 1.55 5.8 ± 1.69
				47.75 ± 2.82					
Leigp 2016	Qidi gushen Decoction (milkvetch root 30–90 g, unprocessed rehmannia root 15–30 g, gordon euryale seed 30–45 g, hedyotis 30–60 g, danshen root 15–20 g, fineleaf schizonepeta herb 10 g)	Prednisone and cyclophosphamide	48/22	38.1 ± 19.7	6	24 h urine protein of less than 0.3 g, serum albumin of more than 35 g/L, normal eGFR.	24 h urine protein of less than 3.5 g, serum albumin improved.	–	6.89 ± 3.31 6.74 ± 2.79
				40.6 ± 22.3					
Leigp 2019	Qidi gushen Tablet (milkvetch root, rehmannia root, gordon euryale seed, hedyotis, danshen root, fineleaf schizonepeta herb)	Angiotensin converting enzyme inhibitor/(prednisone and cyclophosphamide)	15/15	51.69 ± 11.31	6	24 h urine protein of less than 0.3 g, serum albumin of more than 35 g/L, normal eGFR.	24 h urine protein of less than 3.5 g, serum albumin improved.	–	3.87 ± 2.02 4.22 ± 1.75
				48.97 ± 11.61					
Leisb 2020	Huangqi Chifeng Decoction (milkvetch root 30 g, cherokee rose fruit 20 g, gordon euryale seed 20 g, peony root 10 g, divaricate saposhnikovia root 10 g, earthworm 10 g, hedyotis 10 g)	Tacrolimus	33/32	68.35 ± 4.22 68.21 ± 4.01	6	24 h urine protein of less than 0.2 g, serum albumin of more than 35 g/L, normal eGFR.	24 h urine protein of less than 2 g, serum albumin improved.	–	6.53 ± 0.31 6.48 ± 0.35
Liangj 2017	Reinforcing spleen and kidney and clearing heat and activating blood-based treatment (milkvetch root, Chinese yam, largehead atractylodes rhizome, gordon euryale seed, Sichuan lovage rhizome, indian bread, unprocessed rehmannia root, cherokee rose fruit, tortoise carapace and plastron, Chinese angelica, earthworm, baical skullcap root)	Prednisone and cyclophosphamide	25/23	48.76 ± 11.78 47.08 ± 14.77	6	24 h urine protein of less than 0.2 g, serum albumin of more than 35 g/L, normal eGFR.	24 h urine protein of less than 3 g, serum albumin improved.	–	6.05 ± 2.05 7.15 ± 2.51
Lidy 2018	Yishen Jianpi Tongluo Decoction (milkvetch root 30 g, prepared rehmannia root 15 g, asiatic cornelian cherry fruit 15 g, indian bread 15 g, largehead atractylodes rhizome 15 g, Chinese angelica 15 g, earthworm 15 g, danshen root 15 g, Sichuan lovage rhizome 12 g, cassia twig 10 g, liquorice root 3 g)	Prednisone and cyclophosphamide	42/42	45.2 ± 6.5 46.4 ± 6.3	3	24 h urine protein of less than 0.2 g, serum albumin of more than 35 g/L, normal eGFR.	24 h urine protein of less than 3 g, serum albumin improved.	–	4.32 ± 0.51 4.26 ± 0.53
Lij 2020	Wenyang Qushi Tongluo Recipe (milkvetch root 30 g, Chinese angelica 15 g, Sichuan lovage rhizome 12 g, safflower 10 g, leech 3 g, cablin patchouli herb 10 g, dried tangerine peel 15 g, cardamon fruit 10 g, largehead atractylodes rhizome 15 g, indian bread 15 g, epimedium herb 15 g)	Prednisone and cyclophosphamide	60/60	44.8 ± 10.56 45.23 ± 10.07	6	24 h urine protein of less than 0.3 g, serum albumin of more than 35 g/L, normal eGFR.	24 h urine protein of less than 3.5 g, serum albumin improved.	–	7.97 ± 0.81 7.93 ± 0.83
Liuhx 2019	Tonifying Kidney and Removing Blood Stasis and Clearing Away Heat (milkvetch root, tangshen, unprocessed rehmannia root, largehead atractylodes rhizome, Chinese yam, indian bread, Chinese angelica, Sichuan lovage rhizome, danshen root, hedyotis, rhubarb root and rhizome)	Cyclosporin A/tacrolimus and angiotensin converting enzyme inhibitor/angiotensin II receptor blocker	29/29	47.41 ± 13.6 46.34 ± 14.38	6	24 h urine protein of less than 0.3 g, serum albumin of more than 35 g/L, normal eGFR.	24 h urine protein of less than 3.5 g, serum albumin improved.	127.68 ± 35.53 138.65 ± 41.88	7.65 ± 2.47 8.04 ± 2.58
Liuxy 2018	Huatan Quyu Decoction (bile arisaema 10 g, mustard 10 g, tangle 10 g, danshen root 15 g, twotoothed achyranthes root 15 g, motherwort herb 10 g, peony root 10 g, Chinese angelica 15 g, Sichuan lovage rhizome 15 g, largehead atractylodes rhizome 15 g, stiff silkworm 10 g, milkvetch root 30 g)	Prednisone and cyclophosphamide	40/40	44.87 ± 10.16 45.21 ± 9.42	6	24 h urine protein of less than 0.3 g, serum albumin of more than 35 g/L, normal eGFR.	24 h urine protein of less than 3.5 g, serum albumin improved.	–	5.44 ± 2.33 5.1 ± 2.1
Liyg 2020	Shenqizhilong Decoction (hedyotis 30 g, glabrous greenbrier rhizome 15 g, giant knotweed rhizome 15 g, turmeric 10 g, earthworm 10 g, leech 6 g, milkvetch root 20 g, tangshen 20 g)	Tacrolimus	42/42	49.75 ± 6.29 49.82 ± 6.34	3	24 h urine protein of less than 0.3 g, serum albumin of more than 35 g/L, normal eGFR.	24 h urine protein of less than 3.5 g, serum albumin improved.	–	4.25 ± 0.51 4.18 ± 0.53
Loucl 2019 (cyclophosphamide)	Jianpi Yiqi Qingre Huoxue decoction (milkvetch root, tangshen, largehead atractylodes rhizome, radix salviae miltiorrhizae, chinese angelica, motherwort herb, hedyotis, baical skullcap root, plantain herb, coix seed, zhuling)	Prednisone and cyclophosphamide	20/20	54.3 ± 13.3	6	24 h urine protein of less than 0.3 g, serum albumin of more than 35 g/L, normal eGFR.	24 h urine protein of less than 3.5 g, serum albumin improved.	–	7.17 ± 2.23 6.20 ± 2.82
Loucl 2019 (cyclosporin A)	Jianpi Yiqi Qingre Huoxue decoction (milkvetch root, tangshen, largehead atractylodes rhizome, radix salviae miltiorrhizae, chinese angelica, motherwort herb, hedyotis, baical skullcap root, plantain herb, coix seed, zhuling)	Prednisone and cyclosporin A	20/20	49.5 ± 13.6	6	24 h urine protein of less than 0.3 g, serum albumin of more than 35 g/L, normal eGFR.	24 h urine protein of less than 3.5 g, serum albumin improved.	–	6.37 ± 2.43 6.52 ± 2.41
Maxg 2017	Qidi gushen Decoction (milkvetch root 50–90 g, gordon euryale seed 30–45 g, hedyotis 30–60 g, danshen root 15–20 g, fineleaf schizonepeta herb 10 g)	Prednisone/methylprednisolone and cyclophosphamide/tacrolimus	15/14	52.555	6	24 h urine protein of less than 0.3 g, serum albumin of more than 35 g/L, normal eGFR.	24 h urine protein of less than 3.5 g, serum albumin improved.	–	5.47 ± 1.2 5.76 ± 1.08
Shenxm 2017	Jianpi Bushen Prescription (milkvetch root 30 g, epimedium herb 15 g, morinda root 15 g, dodder seed 15 g, indian bread 15 g, largehead atractylodes rhizome 12 g, Chinese yam 15 g, liquorice root 6 g)	Prednisone and cyclophosphamide	30/30	41.00 ± 6.10 42.63 ± 6.31	3	24 h urine protein of less than 0.4 g, serum albumin of more than 35 g/L, normal eGFR.	24 h urine protein of less than 3.5 g, serum albumin improved.	–	4.5 ± 0.553 4.61 ± 0.605
Wangql 2016	Shenqi Dihuang Decoction加减 (tangshen 15 g, milkvetch root 45 g, prepared rehmannia root 15 g, Chinese yam 12 g, asiatic cornelian cherry fruit 15 g, indian bread 15 g, oriental waterplantain rhizome 12 g, tree peony root bark 15 g, golden thread 6 g, danshen root 12 g, Sichuan lovage rhizome 15 g, Chinese magnoliavine fruit 15 g, liquorice root 6 g)	Prednisone and tacrolimus	30/30	47.67 ± 14.812 48.9 ± 16.164	6	24 h urine protein of less than 0.2 g, serum albumin of more than 35 g/L, normal eGFR.	24 h urine protein of less than 3 g, serum albumin improved.	–	5.26 ± 2.1 5.01 ± 1.42
Wangt 2017	Yishen Tongluo Decoction (milkvetch root 30–60 g, prepared rehmannia root 15 g, asiatic cornelian cherry fruit 15 g, cassia twig 10 g, largehead atractylodes rhizome 15 g, indian bread 15 g, Chinese angelica 15 g, Sichuan lovage rhizome 12 g, danshen root 15 g, earthworm 15 g, scorpion 5 g, liquorice root 3 g)	Prednisone and cyclophosphamide	39/36	45.38 ± 6.74 43.59 ± 8.62	3	24 h urine protein of less than 0.2 g, serum albumin of more than 35 g/L, normal eGFR.	24 h urine protein of less than 1 g, serum albumin improved.	–	4.96 ± 0.31 4.91 ± 0.24
Weiyj 2019	Qilong Tongshen Recipe (milkvetch root 30 g, earthworm 10 g, Chinese angelica 12 g, Sichuan lovage rhizome 12 g, peony root 12 g, peach seed 12 g, safflower 12 g, hawthorn 30 g, leech 3 g)	Prednisone and cyclophosphamide	57/57	52.81 ± 9.22 52.84 ± 10.47	3	24 h urine protein of less than 0.3 g, serum albumin of more than 40 g/L, normal eGFR.	Sustained 25–50% reduction in urinary protein.	–	4.76 ± 1.25 4.87 ± 1.48
Wuj 2021	Qidi Gushen recipe (milkvetch root 60 g, raw land 30 g, gordon euryale seed 30 g, hedyotis 30 g, radix salviae miltiorrhizae 20 g, fineleaf schizonepeta herb 10 g)	Prednisone and cyclophosphamide	49/49	52.38 ± 3.76 53.69 ± 3.63	6	24 h urine protein of less than 0.3 g, serum albumin of more than 35 g/L, normal eGFR.	24 h urine protein of less than 3.5 g, serum albumin improved.	–	3.8 ± 1.13 3.78 ± 1.06
Wuqf 2017	Buqi Qufeng Method (milkvetch root 30 g, largehead atractylodes rhizome 10 g, Chinese yam 10 g, asiatic cornelian cherry fruit 10 g, scorpion 4 g, stiff silkworm 10 g, orientvine vine 15 g, cicada slough 10 g, hedyotis 30 g, plantain herb 30 g, peach seed 10 g, safflower 10 g, ground beetle 10 g, Sichuan lovage rhizome 15 g)	Prednisone and cyclophosphamide and tacrolimus	15/15	45.47 ± 15.13 47.53 ± 14.46	6	Proteinuria remained negative.	Sustained 40% reduction in urinary protein, normal eGFR.	–	6.82 ± 2.06 5.91 ± 2.1
Xiex 2018	Tripterygium wilfordii polyglycosides, Yiqi Huoxue Lishui Therapy (milkvetch root 30 g, peony root 20 g, Chinese angelica 20 g, Sichuan lovage rhizome 20 g, peach seed 20 g, largehead atractylodes rhizome 20 g, indian bread 20 g, liquorice root 10 g)	Prednisone and cyclophosphamide	90/90	46.1 ± 1.2 45.4 ± 1.5	6	24 h urine protein of less than 0.3 g, serum albumin of more than 35 g/L, normal eGFR.	Sustained 25% reduction in urinary protein, normal eGFR.	–	6.33 ± 1.56 6.35 ± 1.47
Yangy 2021	Huangtu Yishen Granules (milkvetch root 30–60 g, dodder 15–25 g, cherokee rose fruit 15–30 g, gordon euryale seed 30–50 g, largehead atractylodes rhizome 10–15 g, peony root 10–15 g, chinese angelica 10–15 g, glabrous greenbrier rhizome 20–30 g, oriental waterplantain rhizome 10–15 g)	Prednisone and tacrolimus	21/21	51.10 ± 9.94 52.71 ± 10.45	6	24 h urine protein of less than 0.3 g, serum albumin of more than 35 g/L, normal eGFR.	24 h urine protein of less than 3.5 g, serum albumin improved.	80.99 ± 4.92 81.17 ± 7.06	7.45 ± 2.25 7.68 ± 2.14
Yangyc 2016	Yishen Xiaobai Recipe (milkvetch root 45 g, unprocessed rehmannia root 24 g, largehead atractylodes rhizome 9 g, scorpion 9 g, danshen root 30 g, indian bread 20 g, cicada slough 20 g, stiff silkworm 20 g, earthworm 20 g, leech 5 g, Chinese angelica 15 g, safflower 15 g, peach seed 15 g, Sichuan lovage rhizome 15 g)	Prednisone and cyclophosphamide	33/33	44 vs 42	12	24 h urine protein of less than 0.3 g, serum albumin of more than 30 g/L, normal eGFR.	Sustained 50% reduction in urinary protein, normal eGFR.	–	5.61 ± 3.66 5.49 ± 3.51
Yangzh 2019	Nephrotic Prescription No.1 (milkvetch root 30 g, prepared rehmannia root 20 g, largehead atractylodes rhizome 10 g, divaricate saposhnikovia root 6 g, asiatic cornelian cherry fruit 10 g, dodder seed 15 g, leech 3 g, coix seed 20 g)	Prednisone and cyclophosphamide	39/39	56.92 ± 7.04 56.75 ± 7.12	12	24 h urine protein of less than 0.3 g, serum albumin of more than 35 g/L, normal eGFR.	24 h urine protein of less than 3.5 g, serum albumin improved.	–	7.93 ± 0.72 7.85 ± 0.76
Yux 2018	Yiqi Huoxue Huayu method (milkvetch root 30 g, Chinese yam 30 g, indian bread 20 g, platycodon root 20 g, danshen root 20 g, tree peony root bark 10 g, rehmannia root 20 g, yerbadetajo herb 20 g, gordon euryale seed30 g, cherokee rose fruit 20 g, oyster shell 20 g, Chinese thorowax root 6 g)	Prednisone/methylprednisolone and cyclophosphamide/tacrolimus	15/15	42.33 ± 17.59 47.13 ± 14.11	6	Proteinuria remained negative.	24 h urine protein of less than 3.5 g, serum albumin improved.	–	6.06 ± 1.82 6.71 ± 2.08
Yuy 2015	ShenQi Decoction (milkvetch root 30 g, tangshen 20 g, Chinese yam 20 g, largehead atractylodes rhizome 15 g, prepared rehmannia root 15 g, indian bread 20 g, cassia twig 15 g, danshen root 20 g, leech 5 g, hedyotis 20 g, Chinese magnoliavine fruit 15 g, liquorice root 15 g)	Tacrolimus	28/26	45.5 ± 9.42 46.47 ± 9.12	6	24 h urine protein of less than 0.3 g, serum albumin of more than 35 g/L, normal eGFR.	24 h urine protein of less than 3.5 g, serum albumin improved.	96.31 ± 24.63 93.8 ± 25.1	6.31 ± 2.55 6.36 ± 2.67
Zhangff 2020	Xuantong Sanjiao and Huoxue Tongluo Formula (milkvetch root 45 g, cassia twig 10 g, epimedium herb 10 g, cablin patchouli herb 10 g, dried tangerine peel 12 g, cardamon fruit 10 g, largehead atractylodes rhizome 12 g, indian bread 15 g, Sichuan lovage rhizome 12 g, danshen root 10 g, safflower 12 g, earthworm 12 g, leech 6 g)	Prednisone and cyclophosphamide	30/29	45 vs 42	6	24 h urine protein of less than 0.2 g, serum albumin of more than 35 g/L, normal eGFR.	24 h urine protein of less than 3 g, serum albumin improved.	–	5.86 ± 1.5 5.26 ± 0.92
Zhangyt 2021	Shenqi Dihuang Decoction (heterophylly falsestarwort root, milkvetch root, raw land, chinese yam, asiatic cornelian cherry fruit, indian bread, tree peony root bark, golden thread, radix salviae miltiorrhizae, chinese angelica, sichuan lovage rhizome, hedyotis, liquorice root)	Prednisone and tacrolimus	39/39	50.97 ± 10.42 52.53 ± 11.69	6	24 h urine protein of less than 0.2 g, serum albumin of more than 35 g/L, normal eGFR.	24 h urine protein of less than 3 g, serum albumin improved.	–	7.85 ± 6.09 7.99 ± 6.25
Zhaoc 2019	Yishen Jianpi Huoluo Decoction (milkvetch root 30 g, Chinese yam 30 g, prepared rehmannia root 20 g, largehead atractylodes rhizome 20 g, indian bread 20 g, barbated skullcup herb 15 g, Sichuan lovage rhizome 20 g, danshen root 20 g, leech 10 g)	Prednisone and tacrolimus	40/40	16-66 16-67	6	24 h urine protein of less than 0.4 g, serum albumin of more than 35 g/L, normal eGFR.	24 h urine protein of less than 3 g, serum albumin improved.	–	8.91 ± 1.67 8.78 ± 1.98
Zhaoc 2020	Jianpi Yishen Huoxue Prescription (milkvetch root 30 g, prepared rehmannia root 20 g, Chinese yam 30 g, largehead atractylodes rhizome 30 g, indian bread 30 g, coix seed 30 g, asiatic cornelian cherry fruit 15 g, oriental waterplantain rhizome 10 g, mealy fangji 10 g, Chinese angelica 20 g, peony root 10 g, danshen root 20 g, leech 10 g)	Prednisone and tacrolimus	40/40	45.8 ± 9.8 45.9 ± 9.8	6	24 h urine protein of less than 0.4 g, serum albumin of more than 35 g/L, normal eGFR.	24 h urine protein of less than 3 g, serum albumin improved.	–	9.84 ± 2.89 9.92 ± 2.66
Zhaor 2021	Huangtu Yishen Granules (milkvetch root 30–60 g, dodder 15–25 g, gordon euryale seed 30–50 g, cherokee rose fruit 15–30 g, peony root 10–15 g, chinese angelica 10–15 g, glabrous greenbrier rhizome 20–30 g, largehead atractylodes rhizome 10–15 g, oriental waterplantain rhizome 10–15 g)	Prednisone and tacrolimus	21/20	50.37 ± 11.99 50.07 ± 14.17	6	24 h urine protein of less than 0.3 g, serum albumin of more than 35 g/L, normal eGFR.	24 h urine protein of less than 3.5 g, serum albumin improved.	106.77 ± 16.75 101.97 ± 16.96	6.9269 ± 2.41089 5.78924 ± 1.71299
Zhuyq 2021	Qizhi dihuang decoction (milkvetch root 30 g, leech 6 g, cultivated land 15 g, tangshen 15 g, asiatic cornelian cherry fruit 15 g, chinese yam 15 g, indian bread 15 g, oriental waterplantain rhizome 12 g, tree peony root bark 9 g, debark peony root 12 g, sichuan lovage rhizome 12 g, radix salviae miltiorrhizae 12 g)	Prednisone and cyclophosphamide	31/30	47.27 ± 12.81 49.87 ± 13.32	2	24 h urine protein of less than 0.3 g, serum albumin of more than 35 g/L, normal eGFR.	24 h urine protein of less than 3.5 g, serum albumin improved.	–	7.2 ± 4.4 6.67 ± 2.79
Zuojj 2018	Qiling Tongluo Formula (milkvetch root 20–50 g, largehead atractylodes rhizome 15 g, indian bread 15–30 g, tortoise carapace and plastron 15 g, cicada slough 10 g, black-tail snake 10 g, stiff silkworm 10 g, earthworm 12 g, ground beetle 10 g, leech 3 g, scorpion 3 g, orientvine vine 15 g, glossy ganoderma 15 g)	Methylprednisolone and cyclophosphamide	40/40	48	6	24 h urine protein of less than 0.3 g, serum albumin of more than 35 g/L, normal eGFR.	Sustained 50% reduction in urinary protein, normal eGFR.	–	6.06 ± 1.63 5.96 ± 1.76

### 2.4. Data synthesis and statistical analysis

Data were pooled in Cochrane systematic review software Review Manager (Version 5.4.1) and Stata (Version 12.0) by using a random-effects model when *I*^2^ was greater than 50%. Otherwise, a fixed-effects model was analyzed.^[31]^ Dichotomous data were expressed as odds ratio with 95% confidence intervals (CI). For continuous scales of measurement, results were accessed as mean difference (MD) or standardized mean difference (SMD) with 95% CI. We carried out outcomes analysis based on randomized participants. Where multiple intervention groups in one study existed, pair-wise comparisons related to our study were made.

## 3. Results

### 3.1. Study selection

**Figure 1.**
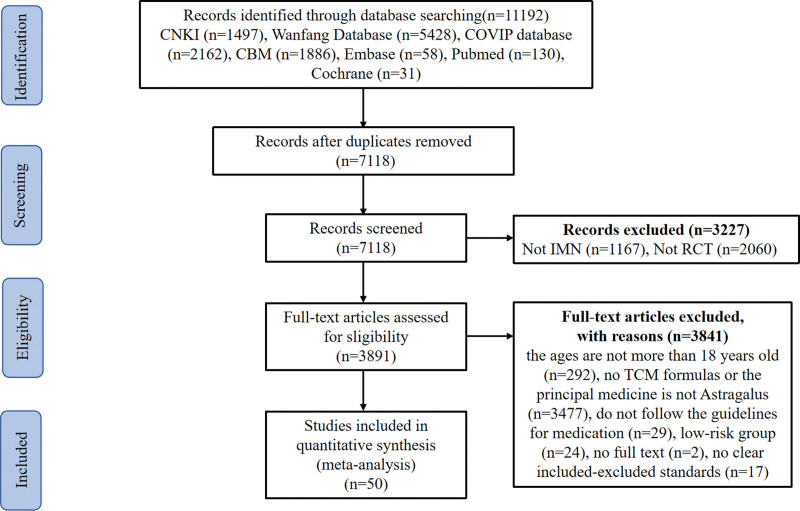
The study flow diagram. CBM = China Biomedical Literature Database, CNKI = China National Knowledge Infrastructure, CQVIP = Chongqing VIP, IMN = Idiopathic membranous nephropathy, RCTs = randomized controlled trials, TCM = Traditional Chinese Medicine.

Eleven thousand one hundred and ninety-two citations were retrieved from the databases. We excluded 4074 duplicates and 3227 irrelevant records. Finally, after screening, 50 eligible studies were identified. The major reason for exclusion were classified as no TCM formulas or the principal medicine is not Astragalus, non-randomized controlled study design, or not IMN. (Fig. 1).

### 3.2. Study characteristics

The characteristics of study were presented in Table 1. All included studies were carried out in China. A total of 3423 participants with IMN were enrolled in the 50 eligible studies.^[32–81]^ Forty eight studies^[32–47,49–60,62–81]^ reported participants’ gender (male 1729 vs female 1279, respectively). Participants’ age ranged from 16 to 68, with a mean age of 48 years old. Ehrenreich-Churg Stage I to IV lesions of MN varied among participants. Fifty studies recruited the two groups of patients. Treatment duration ranged from 1 to 18 months, most of which was 6 months (31/50, 62%). Fifty studies were parallel arm studies, including three study arms. *A membranaceus* preparation was administered with the form of decoction in all studies (50 RCTs). The dose of *A membranaceus* varied from 20 to 90 g/d, and 30 g was the most common dose. *A membranaceus* preparations also included tablet form granule and capsule. Most of the studies reported at least one laboratory outcome of complete remission rate, partial remission rate, proteinuria, or kidney function. None of them measured the endpoint event of mortality or number of patients progressing to ESKD. In all studies, we regarded Huangqi as the principal medicine for its properties consistent with the main aims of the prescription.

### 3.3. Quality assessment

**Figure 2.**
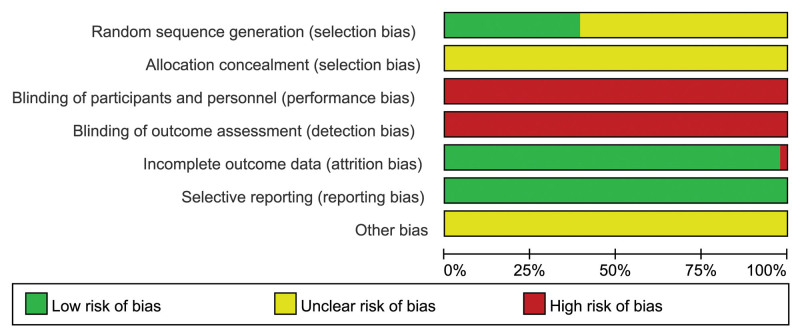
Risk of bias summary of included studies. “?” = unclear risk of bias; “–” = low risk of bias; “+” = high risk of bias.

**Figure 3.**
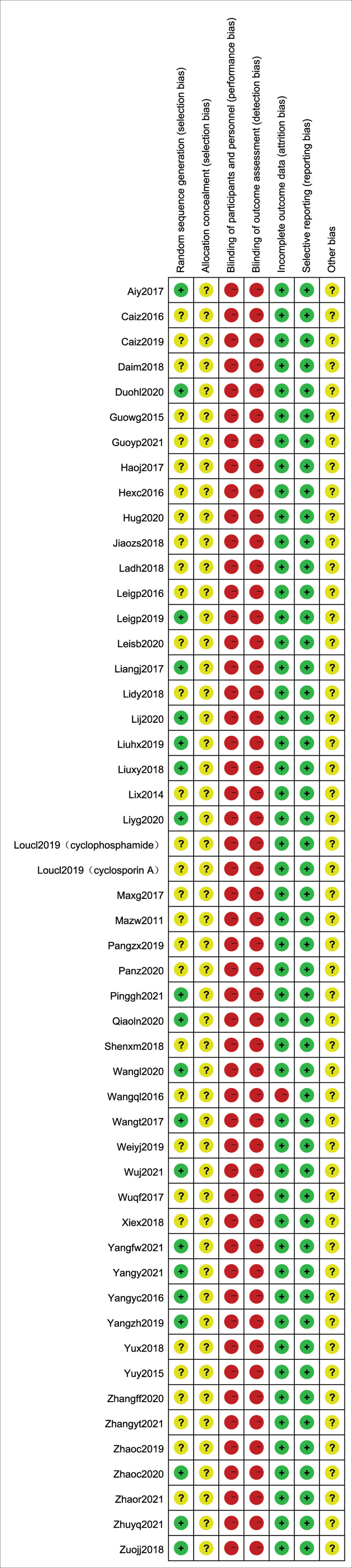
Evaluation for bias risk of included studies.

The methodological quality of literature was present in Figures 2 and 3. All of the included studies mentioned “randomized,” just 20 trials^[37,38,40,43,46,51,54,56,58,59,62,64–66,70,76,78–81]^ provided the details of randomization methods. No eligible studies used blinding methods to decrease performance bias, so all of them were judged as “high risk.” None of them reported allocation concealment. All of them were evaluated at low risk of bias for blinding of outcome assessment in consideration of laboratory test outcomes. All the studies were evaluated as a “low risk” about reporting bias for all the outcome data were reported. Over 90% of studies were judged at low risk of attrition bias because of no withdrawals or losses to follow-up during the study period. One trial^[35]^ reported dropouts, and all provided detailed and reasonable explanations.

### 3.4. Effects on complete response rate

**Figure 4.**
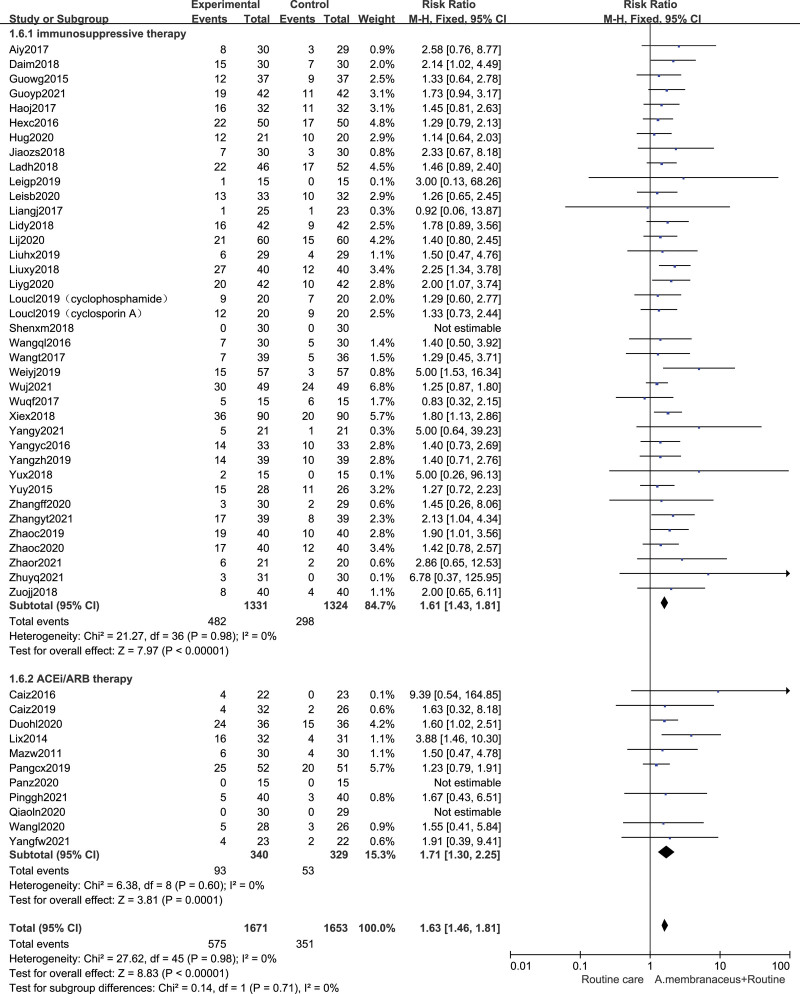
Forest plot of complete response rate. ACEi = angiotensin converting enzyme inhibitor, ARB = angiotensin receptor blocker, CI = confidence intervals.

Complete renal remission was measured in 48 studies^[32,33,35–40,42–81]^ (n = 3324). For moderate-high risk patients, the complete remission of using an oral *A membranaceus* preparation were significantly higher than those without the oral *A membranaceus* preparation (48 trials, n = 3324, RR 1.63 [95% CI 1.46, 1.81]). Further, we performed the subgroup analyses. The pooled estimation indicated that the rates of overall remission in the oral *A membranaceus* preparation combined with nonimmunosuppressive therapy group were significantly higher than those in the nonimmunosuppressive therapy group (11 trials,^[71–81]^ n = 669, RR 1.71 [95% CI 1.30, 2.25]). And the rates of complete remission in the oral *A membranaceus* formula combined with immunosuppressive therapy group were significantly higher than those in the immunosuppressive therapy group alone (37 trials,^[32,33,35–40,42–70]^ n = 2655, RR 1.61 [95% CI 1.43, 1.81]) (Fig. 4).

### 3.5. Effects on partial response rate

**Figure 5.**
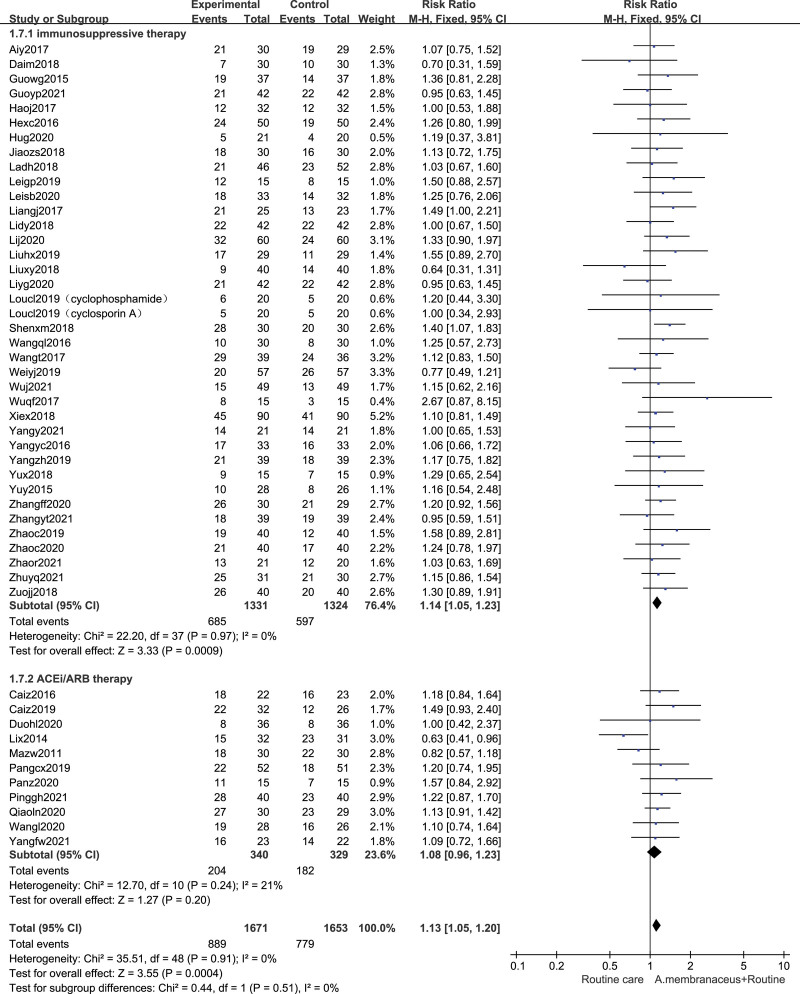
Forest plot of partial response rate. ACEi = angiotensin converting enzyme inhibitor, ARB = angiotensin receptor blocker, CI = confidence intervals.

Forty eight eligible trials^[32,33,35–40,42–81]^ reported partial response rate data (n = 3324). For moderate-high risk patients, the partial remission of using an oral *A membranaceus* preparation were slightly higher than those without the oral *A membranaceus* preparation (48 trials, n = 3324, RR 1.13 [95% CI 1.05, 1.20]). We performed the subgroup analyses. Pooled results indicated that compared with nonimmunosuppressive therapy group alone, the partial remission rate of using Astragalus formula combined were no significantly high (11 trials,^[71–81]^ n = 669, RR 1.08 [95% CI 0.96, 1.23]). The partial remission rate of using Astragalus formula combined with immunosuppressive therapy group were significantly higher than those with immunosuppressive therapy alone (37 trials,^[32,33,35–40,42–70]^ n = 2655, RR 1.14 [95% CI 1.05, 1.23]) (Fig. 5).

### 3.6. Effects on proteinuria

**Figure 6.**
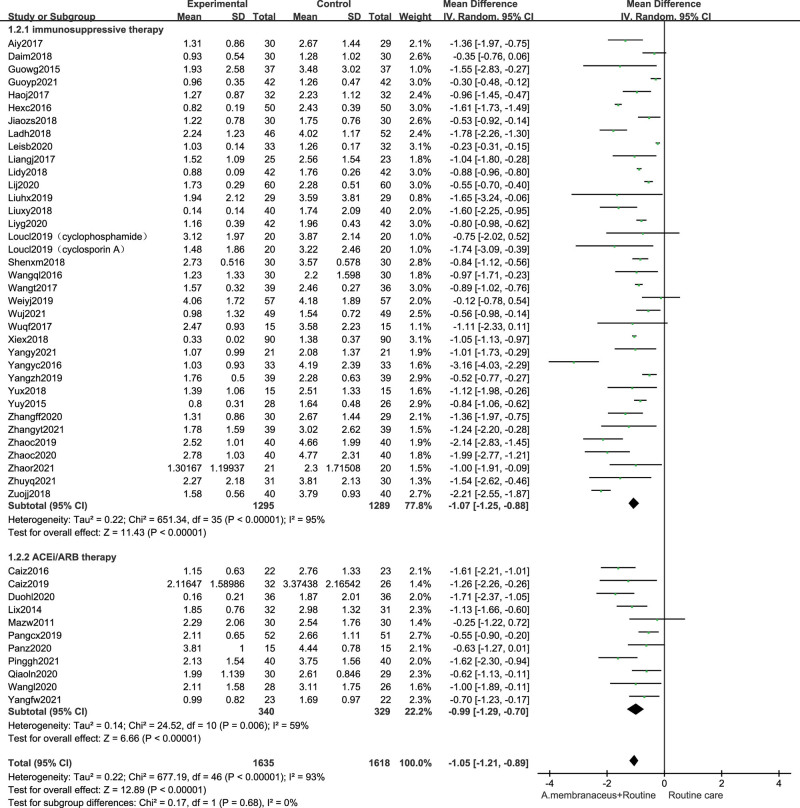
Forest plot of proteinuria. ACEi = angiotensin converting enzyme inhibitor, ARB = angiotensin receptor blocker, CI = confidence intervals, SD = standard deviation.

For moderate-high risk patients, Astragalus preparation significantly decreased 24 hours proteinuria at end of treatment (46 trials,^[32,33,35–40,42–53,55–60,62–81]^ n = 3253, MD −1.05 g/24 h, 95% CI −1.21, −0.89; *I*^2^ = 93%). We performed the subgroup analyses. Pooled results indicated that compared with nonimmunosuppressive therapy group alone, the using Astragalus formula combined suggested lower end-of-treatment proteinuria (11 trials,^[71–81]^ n = 669, MD −0.99 g/24 h, 95% CI −1.29, −0.70; *I*^2^ = 59%). The 24 hours proteinuria level of using Astragalus formula combined with immunosuppressive therapy group were significantly lower than those without the oral *A membranaceus* preparation group (35 trials,^[32,33,35–40,42–53,55–60,62–70]^ n = 2584, MD −1.07 g/24 h, 95% CI −1.25, −0.88; *I*^2^ = 95%), albeit with substantial heterogeneity (Fig. 6).

### 3.7. Effects on kidney function

**Figure 7.**
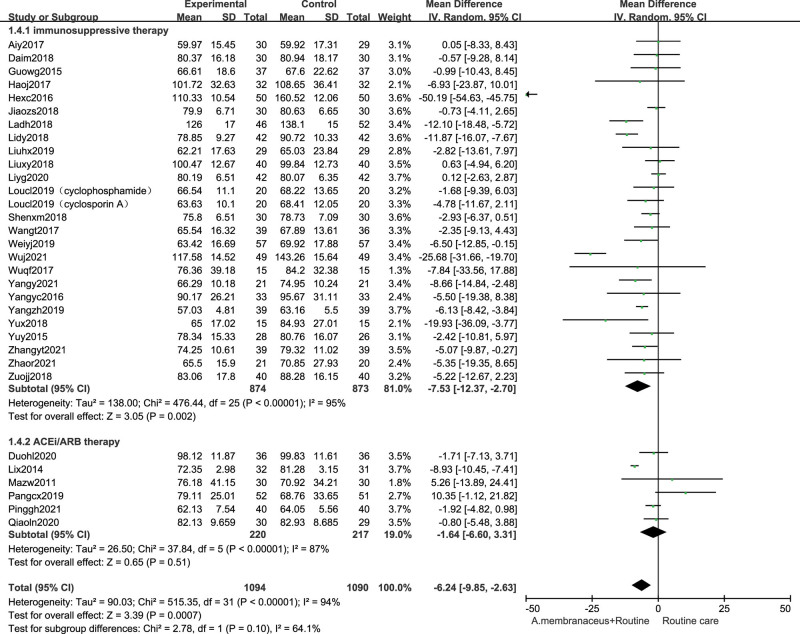
Forest plot of serum creatinine. ACEi = angiotensin converting enzyme inhibitor, ARB = angiotensin receptor blocker, CI = confidence intervals, SD = standard deviation.

For moderate-high risk patients, combining with Astragalus preparation slightly decreased SCr compared with control (31 studies,^[32,33,36–39,42–49,51,52,55–58,64–66,68,69,73–76,78,80]^ n = 2184, MD −6.24 µmol/L, 95% CI −9.85, −2.63; *I*^2^ = 94%). We performed the subgroup analyses. Pooled results indicated that compared with nonimmunosuppressive therapy group alone, the using Astragalus formula combined suggested lower SCr (6 trials,^[73–76,78,80]^ n = 437, MD −1.64 µmol/L, 95% CI −6.60, 3.31; *I*^2^ = 87%). The SCr level of using Astragalus formula combined with immunosuppressive therapy group were significantly lower than those with immunosuppressive therapy alone (25 trials,^[32,33,36–39,42–49,51,52,55–58,64–66,68,69]^ n = 1747, MD −7.53 µmol/L, 95% CI −12.37, −2.70; *I*^2^ = 95%), albeit with substantial heterogeneity (Fig. 7).

### 3.8. Effects on serum albumin

**Figure 8.**
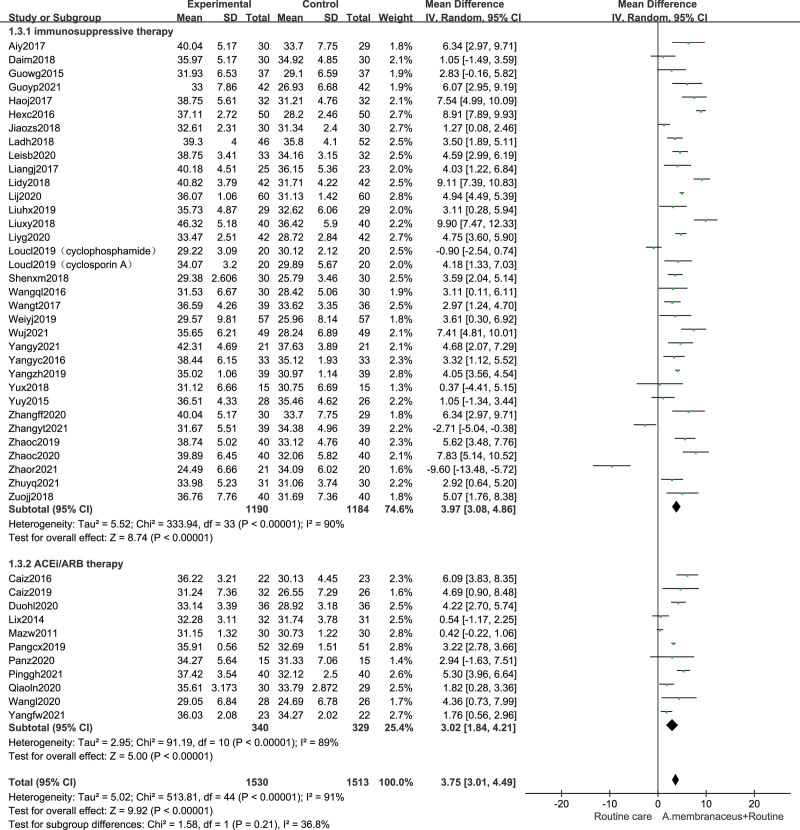
Forest plot of serum albumin. ACEi = angiotensin converting enzyme inhibitor, ARB = angiotensin receptor blocker, CI = confidence intervals, SD = standard deviation.

Forty four trials^[32,33,35–40,43–49,51–53,55–60,62–81]^ measured serum albumin. Immunosuppressive therapy combining with Astragalus preparation significantly improved serum albumin compared with control group (44 studies, n = 3043, MD 3.75, 95% CI 3.01, 4.49; *I*^2^ = 91%). We performed the subgroup analyses. Pooled results indicated that compared with nonimmunosuppressive therapy group alone, the using Astragalus formula combined suggested higher serum albumin (11 studies,^[71–81]^ n = 669, MD 3.02, 95% CI 1.84, 4.21; *I*^2^ = 89%). The serum albumin level of using Astragalus formula combined with immunosuppressive therapy group were significantly higher than those with immunosuppressive therapy alone (33 studies,^[32,33,35–40,43–49,51–53,55–60,62–70]^ n = 2374, MD 4.45, 95% CI 3.08, 4.86; *I*^2^ = 90%), albeit with substantial heterogeneity (Fig. 8).

### 3.9. Safety outcomes

Safety outcomes were reported in 29 trials^[32,33,35,37–41,43–46,48–50,52–59,61–64,69,70]^ out of 50 trials included studies. Ten studies^[35,37,38,44,45,52–54,57,59]^ reported no adverse events occurred during the treatment period. The most common adverse events observed in 19 studies^[32,33,39–41,43,46,48–50,55,56,58,61–64,69,70]^ were gastrointestinal discomfort (74 cases), infect (50 cases), elevated blood sugar (40 cases), abnormal liver function (36 cases), hypertension (26 cases), aleucocytosis (21 cases), Cushing syndrome (17 cases), Insomnia (16 cases). A case of myelosuppression were reported in one studies.^[43]^ All adverse events abated spontaneously and there was no difference for the frequency of adverse events of incidence among the groups.

### 3.10. Publication bias

In point of publication bias, there was suspect in outcomes of complete response rate (Egger’s test *P* = .006), proteinuria (Egger’s test *P* = .002), and SCr (Egger’s test *P* = .015), but not in serum albumin (Egger’s test *P* = .116) and partial response rate (Egger’s test *P* = .986), suggesting a lack of studies with negative results.

## 4. Discussion

This systematic review and meta-analysis was to provide an overview of use of *A membranaceus* preparations in the treatment of moderate-high risk IMN. As antibodies against phospholipase A2 receptor were detected in 70% to 80% of patients, IMN is confirmed as an autoimmune disease now.^[82]^ And then immunosuppressive therapies have been applied to treat IMN patients widely. Among them, rituximab or cyclophosphamide combined with corticosteroids is recommended as a first-line regimen for IMN by the KDIGO guideline.^[83]^ Many studies showed cyclophosphamide combined with corticosteroids has favorable effects in preventing progressing to ESRD.^[84]^ However, the limitation of cyclophosphamide is serious adverse effects associated with accumulated dose.^[85]^ Moreover, cyclosporine and tacrolimus have been effective in promoting remission in 70% of MN patients,^[86]^ but the high rate of relapse of these drugs should not be ignored. More recently, the administration of rituximab has encouraging results. Unfortunately, these biologic agents are too much to afford, especially in less developed Countries. Therefore, we urgently need to find an effective, safe and economic therapeutic method to treat moderate-high risk IMN.

*A membranaceus* is a traditional Chinese medicine with a wide range of active components, mainly including Astragaloside IV, *A membranaceus* polysaccharide and *A membranaceus* isoflavone. It is widely used in clinical practice. There are many extraction preparations of A. membranaceus: *A membranaceus* injection, *A membranaceus* oral liquid, *A membranaceus* capsule, *A membranaceus* polysaccharide for injection and compound *A membranaceus* nasal spray.

More and more evidence show that *A membranaceus* has obvious advantages in the treatment of IMN.^[25,87]^

Some studies on the pharmacological efficacy of *A membranaceus* in treating nephropathy have suggested that it plays an important role in improving renal perfusion, managing blood pressure and delaying renal function progression.^[88]^ But the efficacy of *A membranaceus*-containing formula for moderate-high risk IMN remains to be further reviewed and analyzed.

This meta-analysis included 50 RCTs and involved 3423 patients to evaluating the relationship between *A membranaceus* formula in combination with immunosuppressive therapy and the use of immunosuppressive therapy alone in the treatment of IMN.

Based on the analysis of available data, we found that the efficacy of *A membranaceus* formula combined with immunosuppressive therapy is better than immunosuppressive therapy used alone in the treatment of IMN in improving 24 h UTP, serum albumin, SCr.

### 4.1. Limitations

Evidence on the potential benefits or harms of oral *A membranaceus* preparations for IMN is limited. None of the included studies provided data on calculation of sample size, blindness, allocation concealment and a placebo for the TCM in the control. Most of studies were small and conducted in single hospitals, showing methodological weaknesses especially in terms of randomization. This may have impact on results. The efficacy of oral *A membranaceus* preparations might be overestimated for deficiency of negative studies. *A membranaceus* has been associated with some side effects. However, only 30 studies reported a few adverse effects. Fourth, use of *A membranaceus* preparations may be confined outside East Asia. This poses challenges on the widespread applicability of *A membranaceus* for patients with IMN globally.

## 5. Conclusion

The present review demonstrated that adjunctive use of *A membranaceus* preparations combined with immunosuppressive therapy have a promising treatment for improving complete response rate, partial response rate, serum albumin and reducing proteinuria, serum creatinine levels compared to immunosuppressive therapy in people with MN being at moderate-high risk for disease progression. The overall quality of evidence was low, so the conclusion should be interpreted with caution. There is a need to confirm by high-quality evidence later.

## Author contributions

**Conceptualization:** Lijuan Wang.

**Data curation:** Mingrui Zhang.

**Investigation:** Ping Li.

**Resources:** Dan Wang.

**Supervision:** Qinghua Zhang.

**Visualization:** Dan Wang.

**Writing – original draft:** Dan Wang, Lijuan Wang.

**Writing – review & editing:** Kun Bao.

## Supplementary Material


