# Supplementary material for: Astragalus membranaceus formula for moderate-high risk idiopathic membranous nephropathy: A meta-analysis
Source: Medicine (Baltimore). 2023 Mar 3;102(9):e32918. doi: 10.1097/MD.0000000000032918 (PMC9981402; doi:10.1097/MD.0000000000032918)
Supplement: Supplementary file 1 [file medi-102-e32918-s001.pdf]

Appendix 1

#1: membranous glomerulonephritis OR membranous glomerulopathy OR membranous nephropathy OR extramembranous glomerulopathy OR idiopathic membranous glomerulonephritis OR idiopathic membranous nephropathy OR Heymann

nephritis OR Membranous nephrotic syndrome

#2: Traditional Chinese MedcineOR Chinese Traditional Medicine OR TCM OR Medicine, Traditional OR Chinese Medicine Herb OR Chinese Herbal Drugs OR Chinese Drugs, Plant OR Herbal Medicine OR Herbs OR Alternative Medicine OR Complementary Medicine OR Ethnobotany OR Phytotherapy OR Herbology OR Plants,

Medicinal OR Plant Preparations OR Plant Extracts OR Plants, Medicine OR Materia Medica OR Medicine,Kampo OR Ethnomedicine OR Single Prescription

#3: random control Trial OR random control study OR medical trial OR clinical study OR clinical Trial OR clinical research OR prospective study OR Controlled Trial OR Controlled study OR Multicenter Study OR random allocation OR blinding OR double-blind OR single-blind OR comparative study OR control group OR placebo control OR dummy control OR evaluation study OR follow-up study OR research design OR intervention study OR in vivo study OR case control study

#4: Huangqi OR Beiqi OR Astragalus OR Milkvetch Root OR Milkvetch OR Astragali
